# Supplementary material for: A national survey on availability, price and affordability of selected essential medicines for non communicable diseases in Sri Lanka
Source: BMC Public Health. 2014 Aug 8;14:817. doi: 10.1186/1471-2458-14-817 (PMC4138405; doi:10.1186/1471-2458-14-817)
Supplement: Supplementary file 1 — Additional file 1: List of medicines surveyed. (DOCX 19 KB) [file 12889_2014_6951_MOESM1_ESM.docx]

**SURVEYED MEDICATION LIST**

|  | Name of the medication | Strength | Dosage form | Level of hospital available |
| --- | --- | --- | --- | --- |
| RHEUMATOID DISORDERS | | | | |
|  | Acetylsalicylic acid | 100 mg, 300 mg | Tablet | Level 1, 2, 3, 4 |
|  | Diclofenac Sodium | 50 mg * | Tablet (delayed release) | Level 1, 2, 3, 4 |
|  | Ibuprofen | 400 mg # | Tablet |  |
|  | Paracetamol | 500 mg | Tablet | Level 1, 2, 3, 4 |
|  | Methotrexate | 2.5 mg | Tablet | Level 2, 3, 4 |
| EPELEPSY | | | | |
|  | Carbamazepine | 200 mg | Tablet (Scored) | Level 1, 2, 3, 4 |
|  | Diazepam | 5 mg * | Tablet | Level 1, 2, 3, 4 |
|  | Phenytoin | 100 mg# | Tablet or capsule | Level 1, 2, 3, 4 |
|  | Prednisolone | 5 mg | Tablet | Level 1, 2, 3, 4 |
|  | Sodium valproate | 200 mg/5 ml | Oral liquid | Level 2, 3, 4 |
|  |  | 200 mg | Tablet (enteric coated) | Level 1, 2, 3, 4 |
| PARKINSONISM | | | |  |
|  | Levodopa- carbidopa | 100 mg + 25 mg | Tablet | Level 2, 3, 4 |
| CARDIOVASCULAR DISEASES | | | | |
|  | Atenolol | 50 mg* | Tablet | Level 1, 2, 3, 4 |
|  | Glyceryltrinitrate | 500 μg | Tablet (Sublingual) | Level 1, 2, 3, 4 |
|  | Amlodipine | 5 mg# | Tablet /Capsule | Level 1, 2, 3, 4 |
|  | Amiodarone | 100 mg | Tablet | Level 3, 4 |
|  | Digoxin | 62.5 μg | Tablet | Strength was included in the SL-EML-2009 |
|  | Verapamil | 40 mg | Tablet | Level 3, 4 |
|  | Enalapril | 5 mg# | Tablet | Level 1, 2, 3, 4 |
|  | Captopril | 25 mg* | Tablet/ Capsule | Level 3, 4 |
|  | Hydrochlorothiazide | 25 mg | Tablet (Scored) | Level 1, 2, 3, 4 |
|  | Methyldopa | 250 mg | Tablet | Level 1, 2, 3, 4 |
|  | Nifedipine | 20 mg | Tablet (Prolong release) | Level 1, 2, 3, 4 |
|  | Carvedilol | 6.25 mg | Tablet | Level 3, 4 |
|  | Furosemide | 40 mg | Tablet | Level 1, 2, 3, 4 |
|  | Spironolactone | 100 mg | Tablet | The strength was included in SL-EML-2009 |
|  | Atorvastatin | 10 mg # | Tablet | Level 1, 2, 3, 4 |
|  | Simvastatin | 20 mg* | Tablet/ Capsule |  |
|  | **GASTROINTESTINAL ULCERS AND OTHER DISEASES** | | | |
|  | Famotidine | 20 mg | Tablet | Level 2, 3, 4 |
|  | Omeprazole | 20 mg* | Capsule | Level 2, 3, 4 |
|  | Ranitidine | 150 mg # | Tablet/capsule |  |
| OSTEOPOROSIS | | | |  |
|  | Alendronic acid | 70 mg | Tablet | Level 4 |
| DIABETES | | | | |
|  | Glibenclamide | 5 mg* | Tablet | Level 1, 2, 3, 4 |
|  | Insulin (soluble) | 100 IU/ml in 10 ml vial | Injection | Level 2, 3, 4 |
|  | Intermediate acting insulin | Biphasic (soluble 30% + Amorphous long acting 70%) human insulin 10 IU/ ml in 10 ml vial | Injection | Level 2, 3, 4 |
|  | Metformin | 500 mg # | Tablet | Level 1, 2, 3, 4 |
|  | Gliclazide | 80 mg # | Tablet/ capsule | Level 3, 4 |
| THYROID GLAND DISEASES | | | |  |
|  | Levothyroxine | 100 µg | Tablet | Level 1, 2, 3, 4 |
|  | Carbimazole | 5 mg | Tablet | Level 2, 3, 4 |
| GLAUCOMA | | | |  |
|  | Timolol | 0.25% | Solution (eye drop) | Level 3, 4 |
| PSYCHOTIC DISORDERS | | | |  |
|  | Amitriptyline | 25 mg* | Tablet | Level 1, 2, 3, 4 |
|  | Fluoxetine | 20 mg# | Tablet/Capsule | Level 1, 2, 3, 4 |
|  | Lithium carbonate | 300 mg | Tablet/Capsule | Strength was included in SL-EML-2009 |
| ASTHMA/COPD | | | | |
|  | Beclometasone | 250 µg # | Inhalation (aerosol) | Level 1, 2, 3, 4 |
|  |  | 200 µg | Dry powder capsule | Level 1, 2, 3, 4 |
|  | Ipratropium bromide | 40 µg | Dry powder capsule | Level 1, 2, 3, 4 |
|  | Salbutamol | 100 µg * | Inhalation (aerosol) | Level 1, 2, 3, 4 |
|  |  | 2 mg/5 ml | Oral liquid | Level 1, 2, 3, 4 |
|  |  | 4 mg | Tablet | The strength was included in the SL-EML-2009 |

* = Medicines included in the Global core list = 9

# = Medicines included in the regional core list = 10
